# Refractive shifts in astronauts during spaceflight: mechanisms, countermeasures, and future directions for in-flight measurements

**DOI:** 10.1038/s41433-024-03124-y

**Published:** 2024-05-17

**Authors:** Kelsey Vineyard, Joshua Ong, Benjamin Soares, Daniela Osteicoechea, Cihan Mehmet Kadipasaoglu, Ethan Waisberg, Alireza Tavakkoli, Gianmarco Vizzeri, Andrew G. Lee

**Affiliations:** 1 Edward Via College of Osteopathic Medicine, Spartanburg, SC USA; 2 Department of Ophthalmology and Visual Sciences, University of Michigan Kellogg Eye Center, Ann Arbor, MI USA; 3 Boston University Chobanian & Avedisian School of Medicine, Boston, MA USA; 4 Texas A&M School of Medicine, Bryan, TX USA; 5 Department of Ophthalmology, Blanton Eye Institute, Houston Methodist Hospital, Houston, TX USA; 6 Department of Ophthalmology, University of Cambridge, Cambridge, UK; 7 Human-Machine Perception Laboratory, Department of Computer Science and Engineering, University of Nevada, Reno, Reno, NV USA; 8 Department of Ophthalmology, University of Texas Medical Branch, Galveston, TX USA; 9 Center for Space Medicine, Baylor College of Medicine, Houston, TX USA; 10 The Houston Methodist Research Institute, Houston Methodist Hospital, Houston, TX USA; 11 Departments of Ophthalmology, Neurology, and Neurosurgery, Weill Cornell Medicine, New York, NY USA; 12 University of Texas MD Anderson Cancer Center, Houston, TX USA; 13 Department of Ophthalmology, The University of Iowa Hospitals and Clinics, Iowa City, IA USA

**Keywords:** Refractive errors, Medical imaging

## Introduction

Spaceflight associated neuro-ocular syndrome (SANS) refers to a unique set of neuro-ophthalmic symptoms associated with long-duration space flight documented in astronauts, both in-flight and post-flight. These notable clinical findings include optic disc edema, posterior globe flattening, chorioretinal folds, retinal nerve fiber layer thickening, and hyperopic refractive shifts [1–3]. These hyperopic refractive shifts were among the first findings of SANS, noted by astronauts aboard the international space station (ISS) in 1989 and likely prior, as a visual change necessitating the use of stronger reading glasses [4, 5]. This led to the introduction of what has been referred to as “Space Anticipation Glasses” used by astronauts to mitigate the anticipated effects of long-term spaceflight on visual acuity [6].

These “Space Anticipation Glasses” first referred to the reading glasses brought aboard for long-duration spaceflight in order to combat the hyperopic shifts seen in astronauts. Such glasses would often have to be of increasing strengths [4, 6]. A more advanced countermeasure was created to allow for increased fine-tuning of the prescription required, called “superfocus adjustable glasses” [7]. Superfocus adjustable glasses work via the addition of a flexible lens closer to the eye than the standard firm lens, allowing for dialed control of the lens shape and therefore increased control of acuity [7]. These glasses are now kept aboard the ISS, helping to mitigate the hyperopic shift associated with SANS.

The neuro-ocular changes associated with SANS provide a large barrier to spaceflight, especially with regards to the subsequent changes in vision. Some astronauts returning to earth have demonstrated persistent ocular changes affecting vision including globe flattening and choroidal folds [2]. These residual refractive errors could pose a barrier to continued space flight for astronauts required to meet visual acuity post-refraction standards of 20/20 for both distance and near vision per the National Aeronautics and Space Administration (NASA) [8].

Of the neuro-ocular findings affecting vision, hyperopic shifts have been partially attributed to the posterior globe flattening observed, namely the shortening of the eye’s axial length, reported in about 16% of astronauts post-flight [6]. In this context, globe flattening refers to the decreased convexity of the posterior globe, driving a hyperopic visual shift due to a subsequently reduced axial length. Macias et al. reported that the mean axial length decrease post long-duration spaceflight was 0.08 mm, a finding that persisted, though improving, for the year following [9].

Using pre- and post-flight MRI data, it has been observed that the greatest degree of posterior globe flattening occurs at the initial post-flight scan and was only partially resolved during the first year post-flight [10]. Furthermore, Mader et al. expands upon this, finding that 3 of the 7 astronauts observed showed persistent globe flattening, some up to 7 years after long distance space flight, as well as an associated hyperopic shift of at least +0.75 diopters that similarly persisted [11]. In a case report, Mader et al. concluded that persistent globe flattening began in-flight and was recorded up to 660 days post-flight for one astronaut [12]. This indicates a persistent, albeit variable nature to the ophthalmic changes observed in SANS.

Though the mechanism of SANS is not yet fully understood, it is postulated that the cephalad and orbital shift of fluid during extended low-gravity exposure along with increased intracranial pressure (ICP) may play a significant role [1, 5, 13]. Radiation from galactic cosmic rays and solar particle events likely also play a role in the development of SANS [14, 15]. A terrestrial disease known as idiopathic intracranial hypertension (IIH) has been discussed as an analog for SANS, as IIH has some similar findings such as optic disc edema (ODE) and increased ICP, though SANS lacks many of the other clinical presentations of IIH [13]. Sibony et al. concluded that the differences in ODE and globe flattening between IIH and SANS indicate a differing mechanism rather than increased ICP alone for SANS, though an increased ICP component cannot be ruled out [16]. The prevailing hypothesis therefore is that SANS is likely a multifactorial consequence of the cephalad fluid shift seen in low-gravity and from increased levels of radiation exposure [17]. From a refractive perspective, understanding the mechanism behind SANS, including the hyperopic shift associated, will hopefully lead to effective countermeasures to preserve vision.

Currently on the ISS, the only in-flight vision testing available is performed via self-reported survey, static visual acuity examination, and Amsler Grid [18]. There are also in-flight capabilities of fundoscopy, 2-D ultrasound, Optical Coherence Tomography (OCT), contrast sensitivity, and tonometry [19]. As of now, there are no in-flight options for refraction, including cycloplegic, manifest, or automatic [20]. In-flight refraction would be a beneficial addition to the ISS with regards to understanding the refractive shifts associated with SANS during spaceflight. In monitoring refractive shifts throughout space flight, future investigations may be able to more closely relate the timeline of SANS clinical findings with the hyperopic refractive shifts detailed in previous research. Furthermore, such measurements would provide more data on visual acuity changes associated with spaceflight related to refractive error, some of which may resolve in-flight and not appear on post-flight evaluation.

When discussing refraction options for the ISS, it is important to first investigate the current terrestrial options available. These technologies must be evaluated for their portability, weight, and accuracy as spaceflight requires uniquely portable and lightweight technology. Durr et al. found that a novel handheld QuickSee wavefront autorefractor was able to, operated by an individual with no formal ophthalmic or optometric training, create a prescription only one letter worse compared to a formal manifest refraction by a trained professional [21, 22]. Ciuffreda et al. determined that an alternative, phone mounted autorefractor called the SVOne showed no significant difference in spherical refractive error between the SVOne and other forms of refraction. This study also determined that the SVOne had a not statistically significant, but present, tendency to overestimate myopia by approximately 0.4 diopters [23]. Research on the Near Eye Tool for Refractive Assessment (NETRA) compared to non-cycloplegic refraction demonstrated a similar overestimation of myopia at 1.25 diopters more in NETRA than in standard refraction [24]. In the discussion of SANS, this overestimation of myopia could pose a barrier to the accurate measurement of established hyperopic shift and would have to be considered.

Agarwal et al. compared the use of the QuickSee, NETRA, and Retinomax handheld autorefractors, stating limitations of a high amount of delicacy and cost for the Retinomax and lower accuracy comparatively for the NETRA as well as the requirement of an android smartphone for attachment [25]. These factors could be unideal in the context of spaceflight refraction.

Another consideration for refraction options for spaceflight includes the recognition of space as an austere environment with unique challenges that technology must meet [26, 27]. Joseph et al. described the use of a handheld lightweight, non-invasive refraction device operated via the adjustment of dials. This refractive device, known as the ClickCheck, demonstrated however that the spherical and cylindrical refractive errors determined were statistically significant in difference from standard refraction and thus more research and development is needed for it to be an alternative to standard subjective refraction methods [28]. The ClickCheck or similar technology could provide an inexpensive and highly portable refraction technique for remote areas without ample resources.

Of interest for in-flight refraction is a head-mounted refraction technique such as with virtual reality (VR). Pujol et al. evaluated an experimental VR based algorithm headset for subjective refraction, demonstrating notable precision in spherical equivalency [29]. Waisberg et al. discussed the use of a functional and comparably agreeable head-mounted device for measuring distance visual acuity in spaceflight [30]. This technology, though not directly refractive in nature, demonstrates a successful use of visual measurement using a VR headset in-flight that may be in the future adjustable to the needs of refraction aboard the ISS. However, utilizing near vision targets for refraction may induce time-dependent transient myopic shifts and thus affect the refractive error outcomes in head-mounted devices [31]. These transient shifts may be accounted for within the creation of VR refraction technology.

## Conclusion

SANS and the associated hyperopic refractive shifts with long-duration spaceflight pose distinct challenges for astronauts particularly as space exploration continues to grow over time. Though there currently exists no method of refraction aboard the ISS, VR or other lightweight portable approaches to refraction could provide a unique and multifunctional method through which SANS could be monitored during spaceflight. Further research aimed at refractive methods aboard the ISS may help to provide insight into the mechanism of SANS. There are also numerous potential terrestrial applications of lightweight, portable refractive methods in rural or remote parts of the world that could be derived from such research.
